# Investigation of Spectroscopic Parameters and Trap Parameters of Eu^3+^-Activated Y_2_SiO_5_ Phosphors for Display and Dosimetry Applications

**DOI:** 10.3390/molecules30010108

**Published:** 2024-12-30

**Authors:** Neeraj Verma, Marta Michalska-Domańska, Vikas Dubey, Tirath Ram, Jagjeet Kaur, Neha Dubey, Shireen Aman, Ovica Manners, Janita Saji

**Affiliations:** 1 Department of Physics, Government G.S.G.PG. College, Balod 491226, Chhattisgarh, India; neerajphys14@gmail.com; 2 Department of Physics, Government Vishwanath Yadav Tamaskar Post Graduate Autonomous College, Durg 491001, Chhattisgarh, India; tirathsinha@gmail.com (T.R.); jagjeet_62@yahoo.co.in (J.K.); tiwarineha1441@gmail.com (N.D.); 3 Institute of Optoelectronics, Military University of Technology, 00-908 Warsaw, Poland; 4 Department of Physics, North-Eastern Hill University (NEHU), Shillong 793022, Meghalaya, India; amanshireen123@gmail.com (S.A.); ovicamanners@gmail.com (O.M.); 5 Department of Science and Humanities, School of Engineering and Technology, CHRIST University, Bangalore 560029, Karnataka, India; janita.saji@christuniversity.in

**Keywords:** phosphors, spectroscopic parameters, trap parameters, CGCD, CIE

## Abstract

Using the solid-state reaction technique, varied Y_2_SiO_5_ phosphors activated by europium (Eu^3+^) ions at varied concentrations were made at calcination temperatures of 1000 °C and 1250 °C during sintering in an air environment. The XRD technique identified the monoclinic structure, and the FTIR technique was used to analyze the generated phosphors. Photoluminescence emission and excitation patterns were measured using varying concentrations of Eu^3+^ ions. The optimal strength was observed at a 2.0 mol% concentration. Emission peaks were detected at 582 nm and 589 nm for the ^5^D_0_→^7^F_1_ transition and at 601 nm, 613 nm, and 632 nm for the ^5^D_0_→^7^F_2_ transition under 263 nm excitation. Because Eu^3+^ is naturally bright, these emission peaks show how ions change from one excited state to another. This makes them useful for making phosphors that emit red light for use in optoelectronics and flexible displays. Based on the computed (1931 CIE) chromaticity coordinates for the photoluminescence emission spectra, it was determined that the produced phosphor may be used in light-emitting diodes. The TL glow curve was examined for various doping ion concentrations and durations of UV exposure levels, revealing a broad peak at 183 °C. Using computerized glow curve deconvolution (CGCD), we calculated the kinetic parameters.

## 1. Introduction

Silicate-based phosphors have garnered significant interest due to their strong chemical stability, low thermal expansion, high conductivity, and impressive optical damage threshold [1,2,3,4,5]. Yttrium oxyorthosilicate (Y_2_SiO_5_) is highly regarded as an important framework for rare-earth activators, with rare-earth ions substituting Y^3+^ ions at two distinct crystallographic sites with C_1_ symmetry [6]. Y_2_SiO_5_, when doped with rare-earth elements, demonstrates exceptional luminescent properties, which can be attributed to its enduring chemical and physical stability [7,8,9,10,11]. Previous studies, including research by Zhang et al. (2017), demonstrated a shift in the main emission band of Y_2_SiO_5_:Eu^3+^ to longer wavelengths, making it suitable as a red phosphor for white LEDs [12]. Moreover, the luminescence intensity in Y_2_SiO_5_:Eu^3+^ was enhanced by co-doping with Ge^4+^ ions. The introduction of Ge^4+^ ions into Y_2_SiO_5_:Eu^3+^ can cause the primary emission band to shift towards longer wavelengths, thereby improving the phosphor’s color quality. This discovery suggests that in Y_2_SiO_5_:Ge^4+^, Eu^3+^ has great potential as a red-emitting phosphor for near-ultraviolet (NUV) white LEDs [13]. In 2015, Parganiha and colleagues prepared Y_2_SiO_5_:Eu^3+^ phosphor using the solid-state reaction technique. They noted that this phosphor can serve as the sole host for red emission in display devices and light-emitting diodes (LEDs), and may also have potential applications as a thermoluminescence dosimetric material when exposed to UV light. Additionally, they reported that Y_2_SiO_5_:Ce^3+^ is effective in blue light emission when used in NUV-excited LEDs [14]. Kang et al. 2015 investigated the emissions of Y_2_SiO_5_:Bi^3+^ between blue and green, attributing them to energy transfer between Bi centers. When Eu^3+^ is co-doped in Y_2_SiO_5_:Bi^3+^, energy transfer from Bi^3+^ to Eu^3+^ occurs through electric dipole–dipole interaction [15]. Upadhyay et al. 2023 synthesized YSO: Eu^3+^ phosphor, observing broad excitation peaks at 257 nm with a shoulder at 275 nm, and an emission spectra peaked at 617 nm, confirming strong red emission [16]. Their study focused on synthesizing and characterizing Eu^3+^-activated YSO phosphor with varying dopant concentrations and analyzing its kinetic and spectroscopic parameters.

## 2. Experimental Section

The phosphor of Eu^3+^ ions incorporated into Y_2_SiO_5_ was prepared using the novel solid-state reaction method with varying Eu^3+^ molar concentrations, ranging from 0.1% to 2.5%. The novelty of this method lies in the optimization of different parameters, such as reaction temperature, duration, and mixing of the precursor, in order to acquire enhanced homogeneity and phase purity of the final product. Also, this method is suitable for the large-scale production of phosphors, without compromising the material quality. The essential components for synthesizing Y_2_SiO_5_:Eu^3+^ included Y_2_O_3_, SiO_2_, Eu_2_O_3_, and H_3_BO_3_ (at 0.05 mol%), which served as a flux. The quantity of each chemical was determined according to the stoichiometric ratio, and they were then thoroughly mixed with a mortar and pestle for a duration of two hours. Following the mixing process, the powdered sample was placed in an alumina crucible and exposed to calcination at 1000 degrees Celsius for an hour, and then sintered at 1250 degrees Celsius for three hours in a muffle furnace. After the completion of the heating process, an intermediate grinding stage commenced. The following is the comprehensive reaction:

Y_2_O_3_ + SiO_2_ + Eu2O3 → Y_2_SiO_5_:Eu^3+^

The materials were characterized using photoluminescence, FTIR, thermoluminescence, SEM, EDX, and XRD techniques. A Bruker D8 Advance X-ray diffractometer was utilized for the XRD measurements. The XRD measurements involved the generation of X-rays with a wavelength of 0.154 nm from a tightly sealed tube (Cu Kα). The silicon strip technique was used to detect X-rays, and a Bruker LynxEye detector, a quick counting detector, was utilized for this purpose. Using a Shimadzu RF-5301 PC spectrofluorophotometer, the PL emission and excitation spectra were acquired at room temperature. An excitation source from a xenon lamp was utilized for this purpose. A 1009I TLD reader from Nucleonix Sys. Pvt. Ltd. of Hyderabad, India [17,18,19,20,21,22,23,24] was employed to record glow curves from thermally induced luminescence. For TL analysis, the phosphor was exposed to UV light with a wavelength of 254 nm. The TL measurements utilized a heating rate of 2.5 °C s^−1^.

## 3. Results and Discussions 

### 3.1. XRD Analysis of Y_2_SiO_5_: Eu^3+^

**Figure 1:**
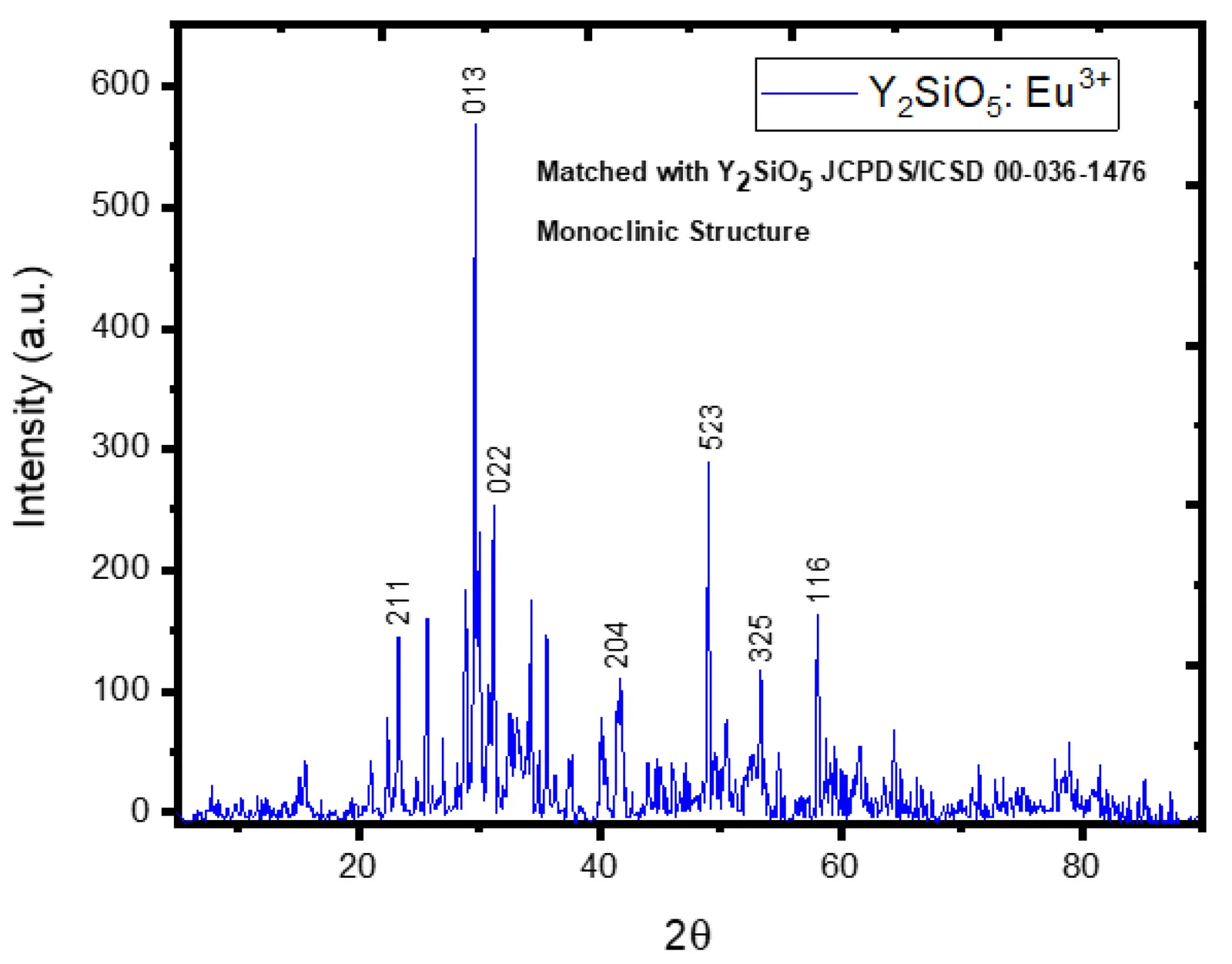
XRD Pattern of Y_2_SiO_5_: Eu^3+^ phosphor (2.0 mol%).

The XRD pattern for the optimized 2.0 mol% Y_2_SiO_5_: Eu^3+^ phosphor concentration is shown in Figure 1. As the size of the crystallites becomes smaller, the peak’s width increases. Scherrer’s formula and the FWHM of the strong peak were used to determine the crystallite size. The following formula was used to achieve the calculation:

$$D=\frac{0.9\lambda }{\beta cos\theta }$$

The crystallite size (D), diffraction angle (θ), X-ray wavelength (λ), and diffraction full width half maximum (FWHM) are all denoted here. The Miller indices were calculated for the XRD pattern, and they were matched to the Standard ICDD card number MP-1179266, as seen in Figure 1. The sample’s structure was Monoclinic. The utilization of Scherrer’s formula revealed a crystallite size of 30.82 nm. Table 1 displays the average crystallite size, strain, and dislocation values derived from the XRD diffraction pattern. The averaging of the crystallite sizes from different planes considering their respective intensities is referred to as the ‘average crystallite size’. This value basically serves as a simplified representation of the overall crystallite size in the material.

### 3.2. FTIR Analysis of Y_2_SiO_5_: Eu^3+^

**Figure 2:**
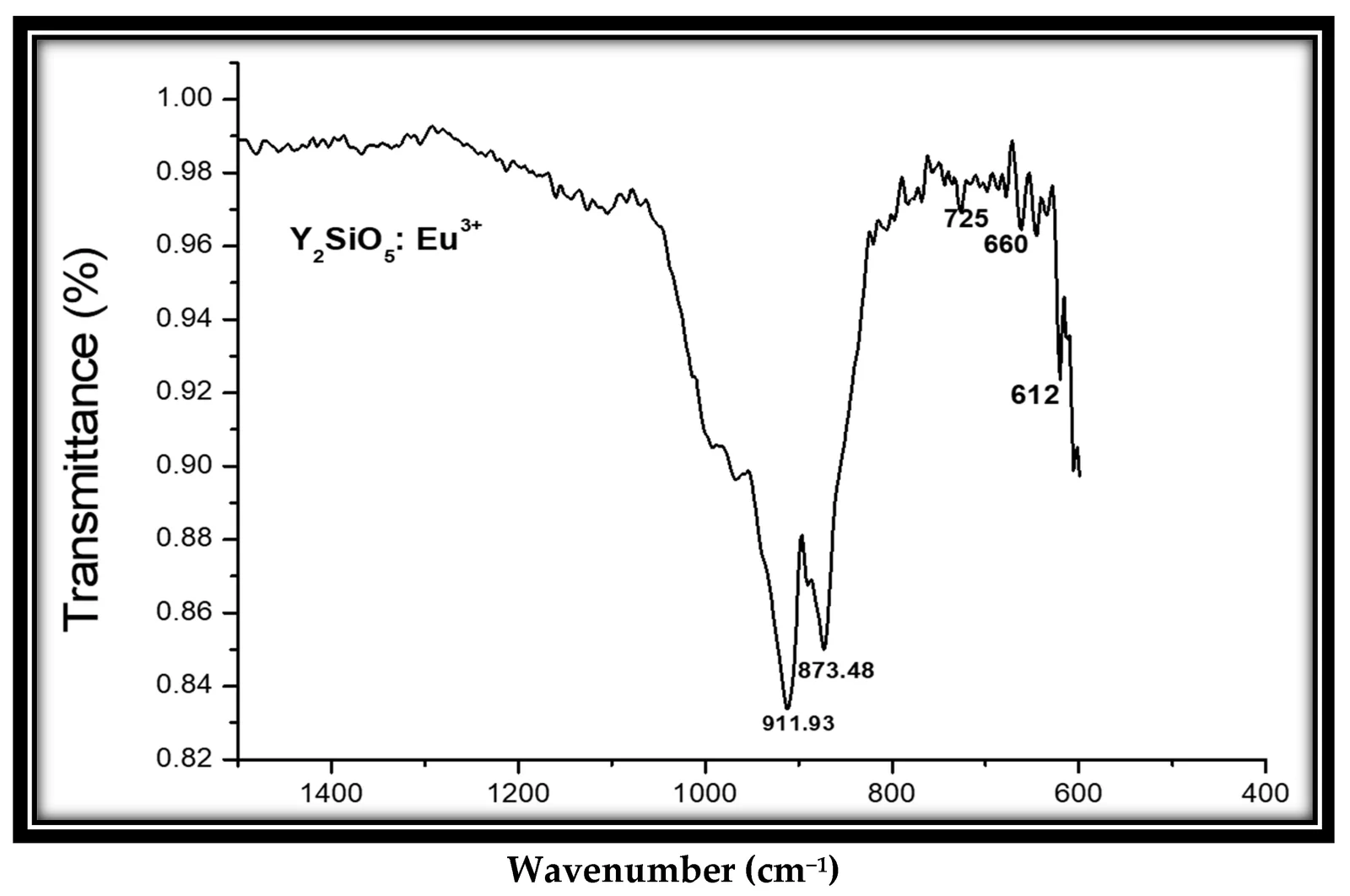
FTIR spectra of Y_2_SiO_5_: Eu^3+^ phosphor (2.0 mol%).

The FTIR spectrum for Y_2_SiO_5_:Eu^3+^ displays distinct absorption characteristics that offer insights into the structural makeup of the material, as shown in Figure 2. The broad range from 1400 to 1000 cm⁻^1^ exhibits little vibrational activity, suggesting that there are no major hydroxyl or organic contaminants. Notable peaks at 911.93 cm⁻^1^ and 873.48 cm⁻^1^ correspond to Si-O-Si asymmetric stretching vibrations, affirming the existence of a silicate framework. Additional peaks at 725 cm⁻^1^, 660 cm⁻^1^, and 612 cm⁻^1^ are linked to lattice vibrations, likely involving interactions between the Eu^3+^ dopants and the surrounding oxygen in the matrix [18]. These features underscore the structural stability of the Y_2_SiO_5_ host lattice and the successful incorporation of Eu^3+^ ions, with no significant impurities observed.

### 3.3. SEM and EDX Analysis of Y_2_SiO_5_: Eu^3+^

**Figure 3:**
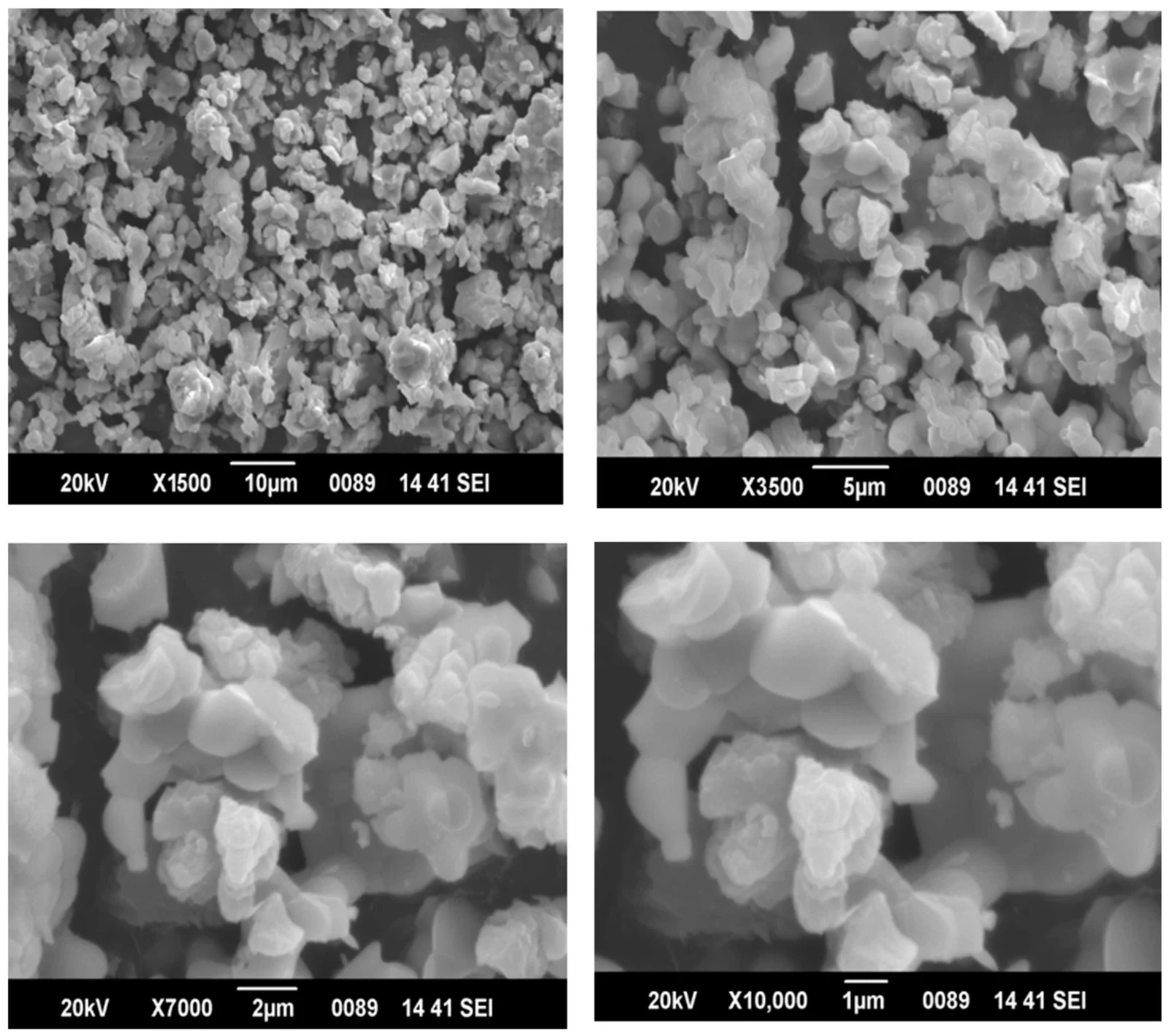
SEM images of Y_2_SiO_5_: Eu^3+^ (2.0 mol%).

The shape and surface characteristics of several phosphors doped with rare-earth elements were examined using a sophisticated characterization method known as scanning electron microscopy (SEM). SEM gives high-resolution pictures of rare earths doped with various phosphors. The approach may also be utilized to monitor any morphological changes caused by the synthesis process or heat treatment. Cracks, pores, and other imperfections in the phosphor particles’ surfaces may be seen using scanning electron microscopy (SEM), which can then be used to optimize the phosphor’s luminosity. SEM images of Y_2_SiO_5_: Eu^3+^ (2.0 mol%) reveal diverse agglomerate shapes, including sponge-like and small spherical shapes, in Figure 3. The particle size is estimated to be a few microns, and the surface morphology appears to be good.

**Figure 4:**
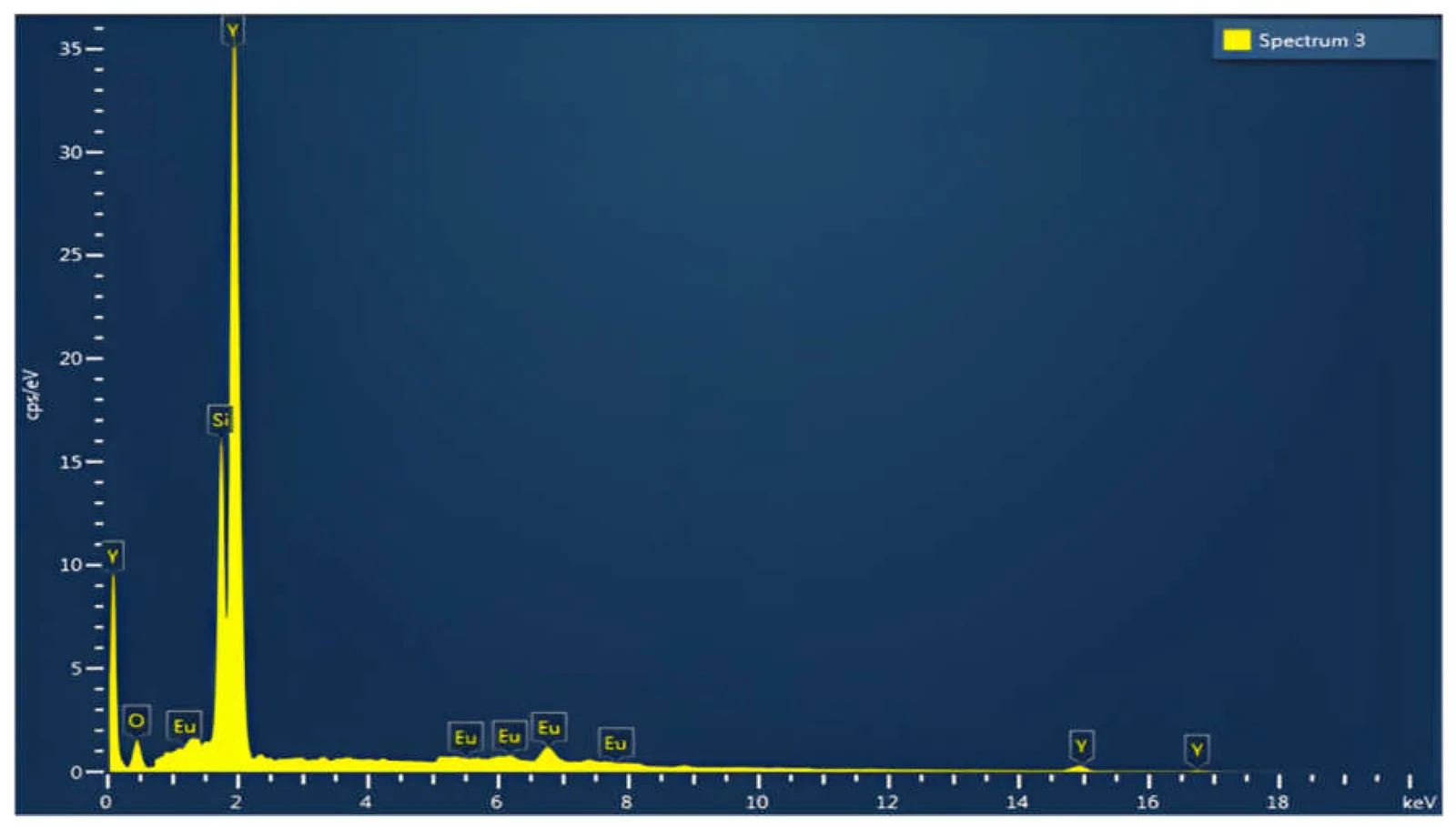
EDX spectra of Y_2_SiO_5_: Eu^3+^ phosphor (2.0 mol%).

Energy-Dispersive X-ray Spectroscopy (EDX) is a useful tool for studying the chemical construction of different phosphors that have been doped with rare earths. It can also give important information about the luminescence qualities of the materials. Using this technology, we may increase the stability and luminescence efficiency of the material by adjusting the phosphor’s elemental composition and concentration. This has major consequences for applications like lighting and sensing. Figure 4 shows EDX spectra which depict the presence of yttrium, silicon, oxygen, and europium atoms in the Y_2_SiO_5_: Eu^3+^ phosphor (2.0 mol%).

The powder mapping EDX spectra of Y_2_SiO_5_: Eu^3+^ phosphor (2.0 mol%) provide insights into the elemental composition, confirming both the stoichiometry of the material and the effectiveness of doping. As shown in Table 2, oxygen (O) is the predominant component, comprising 30.18 wt% and 65.7 atomic%, highlighting its significance in the oxide lattice. Silicon (Si) contributes 8.55 wt% and 10.6 atomic%, affirming the existence of silicate groups. Yttrium (Y) is the most prevalent element by weight at 59.47 wt% and 23.3 atomic%, forming the foundation of the Y_2_SiO_5_ host lattice. Europium (Eu), the dopant, is detected at 1.81 wt% and 0.4 atomic%, consistent with the specified doping level. These findings confirm the effective integration of Eu^3+^ into the phosphor and the overall purity of the material.

### 3.4. Photoluminescence Study of Y_2_SiO_5_: Eu^3+^

**Figure 5:**
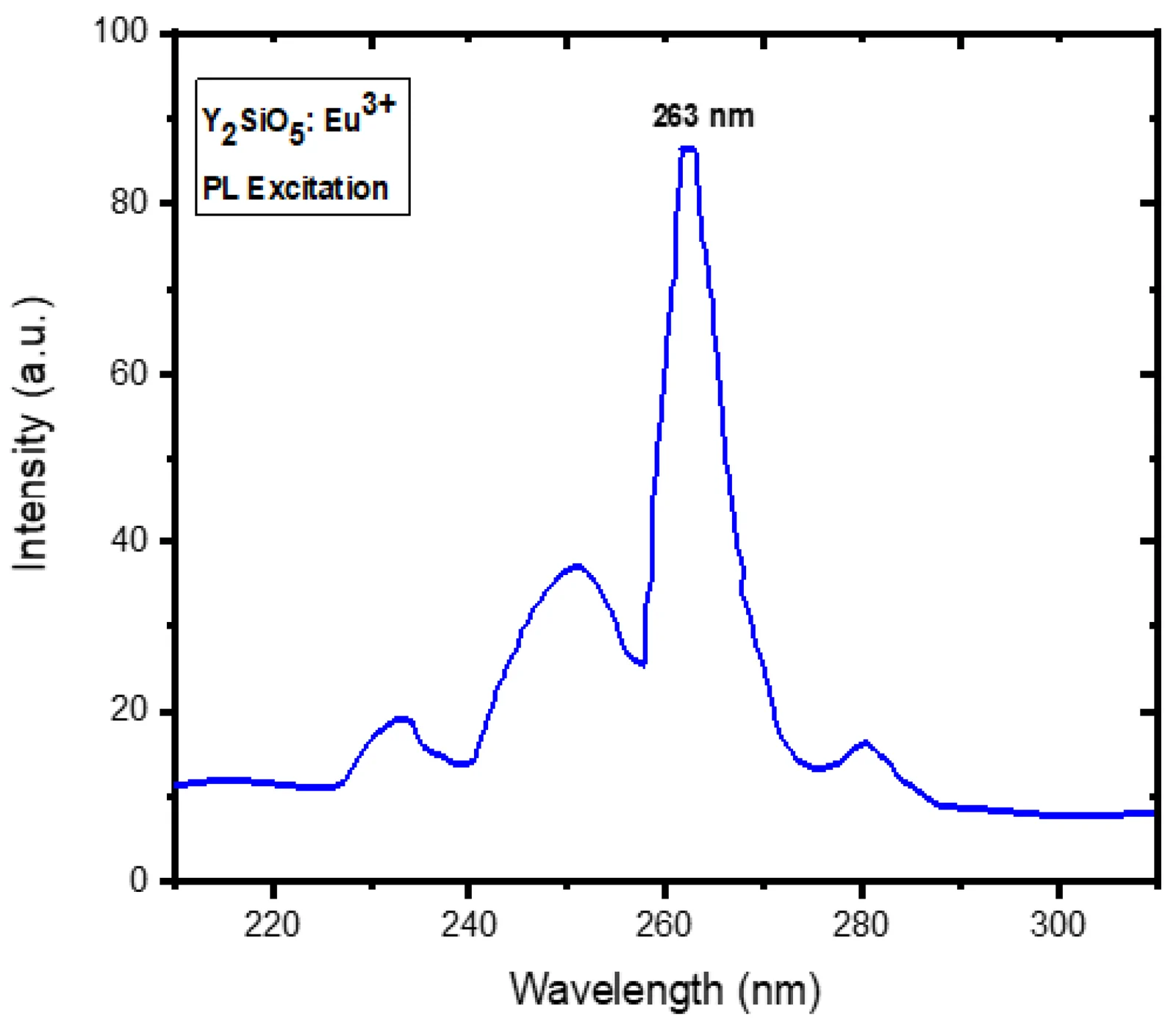
PL excitation spectra of Y_2_SiO_5_: Eu^3+^ phosphor (2.0 mol%).

**Figure 6:**
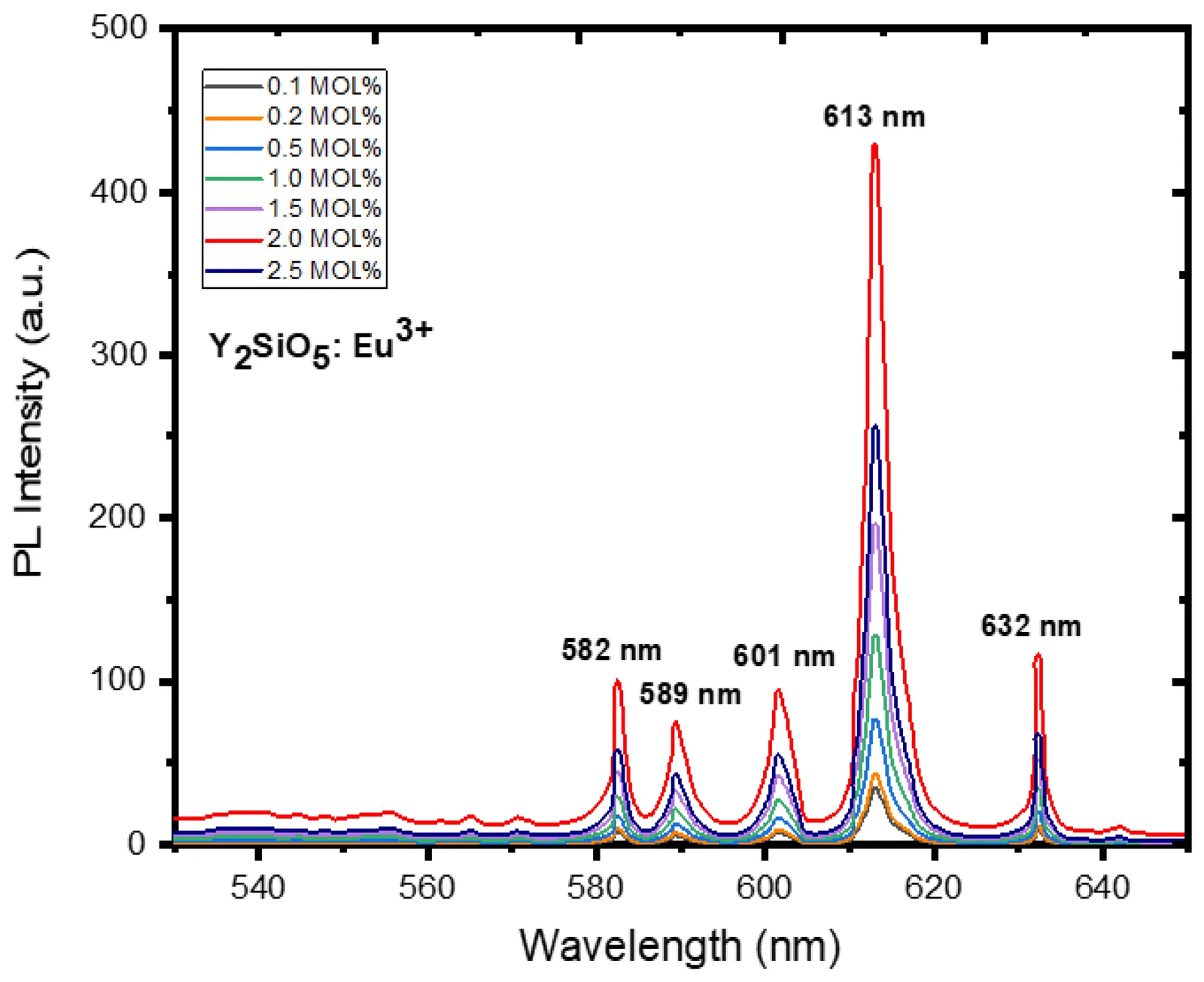
PL emission spectra of Y_2_SiO_5_: Eu^3+^ phosphor for all concentrations.

**Figure 7:**
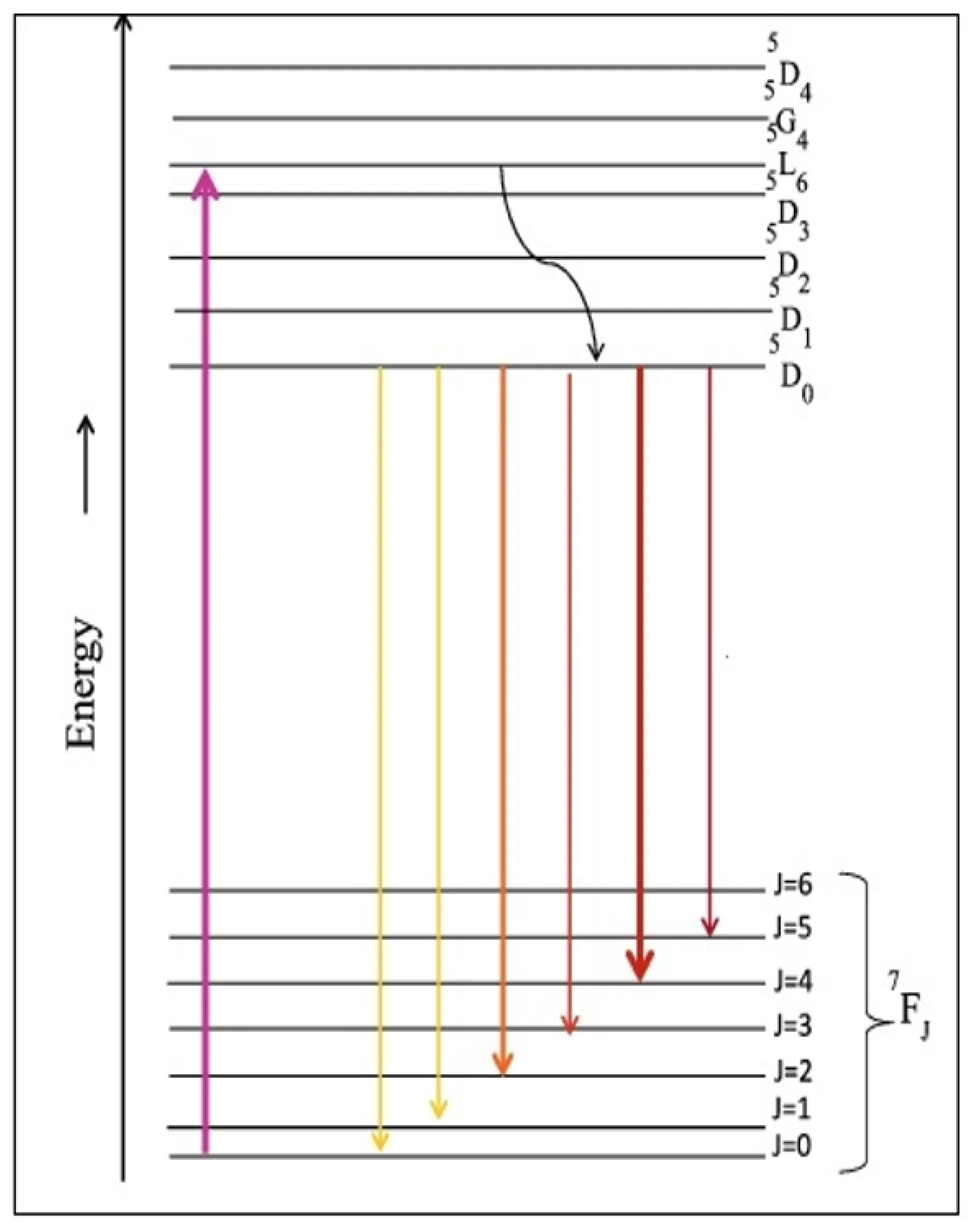
Energy level diagram for Eu^3+^ phosphor.

Incorporating Eu^3+^ ions into the non-luminescent host Y_2_SiO_5_ phosphor significantly enhanced its emission spectrum. The excitation spectra for the photoluminescence (PL) of Eu^3+^-doped Y_2_SiO_5_ phosphor, as depicted in Figure 5, were recorded with an emission wavelength of 263 nm. The selection of excitation and luminescence wavelengths in our study was based on the specific transitions of Eu^3+^ ions in the host matrix under investigation. While Eu^3+^ indeed exhibits prominent luminescence bands around 613–640 nm (corresponding to the hypersensitive transition ^5^D_0_→^7^F_2_) and near 700 nm (attributed to ^5^D_0_→^7^F_4_), the luminescence spectra in the range of 540–650 nm were primarily analyzed to encompass both the ^5^D_1_→^7^F_J_ and ^5^D_0_→^7^F_J_ transitions to achieve a comprehensive understanding of the emission characteristics. Regarding the excitation spectrum recorded at a luminescence wavelength of 263 nm, this was carried out to ensure the identification of the most efficient excitation bands that contribute to the observed emission. The selection of 400 nm as the monitored emission wavelength was influenced by the overlap of weaker emissions related to higher-energy states (^5^D_2_) and their potential influence on energy transfer processes. This approach allowed us to explore the host material’s energy transfer dynamics and sensitization effects in detail. These spectra displayed a wide excitation peak at 263 nm. When excited at this specific wavelength, the photoluminescence (PL) emission spectrum showed clear emissions at 582 nm, 589 nm, 601 nm, 613 nm, and 632 nm. These emissions come from transitions occurring within the Eu^3+^ ions, particularly from the excited state ^5^D_0_ to the ground state ^7^F_J_ (J = 0, 1, 2), as illustrated in Figure 6. The most intense emission is observed at 613 nm, corresponding to the electric dipole transition ^5^D_0_→^7^F_2_ [18]. Other emissions at 582 nm, 589 nm, and 601 nm are attributed to the magnetic dipole transition ^5^D_0_→^7^F_1_, which occurs due to the similar sizes of Eu^3+^ and Y^3+^ ions, allowing Eu^3+^ to substitute Y^3+^ in the lattice [25]. Figure 7 highlights the influence of dopant concentration on the emission intensity of the Y_2_SiO_5_: Eu^3+^ phosphor, emphasizing the significant effect of host composition on luminescence properties. While the emission spectra show small separation of the ^5^D_0_→^7^F_1_ and ^5^D_0_→^7^F_2_ transition lines, the current spectral resolution does not allow for definitive conclusions regarding the influence of crystal structure or coordination environment. The intensity increases from 0.1 to 2.0 mol% as the Eu^3+^ ion concentration increases. However, for 2.5 mol% of the period, the intensity drops drastically due to the concentration quenching event. The movement of Eu^3+^ ions closer together and their subsequent interactions and charge transfer result in a decrease in intensity known as quenching. Additional data on luminescence lifetimes would provide a more comprehensive understanding of the concentration quenching mechanism. Also, other factors, like reabsorption and grain size, may have some impact on the luminescence intensity of the phosphors. Under the given experimental conditions, the maximum emission intensity was observed for 2.0 mol% Eu^3+^-doped Y_2_SiO_5_ phosphors, making them ideal for a variety of light-emitting applications.

### 3.5. (CIE) Coordinates

The color of the light produced by these phosphors is often described using the CIE coordinate system. A three-dimensional space called the CIE color space uses the three variables, X, Y, and Z, to define every conceivable color. Red, green, and blue light are used in varying proportions to produce each color, and these proportions are represented by the X, Y, and Z coordinates. The CIE color space provides a useful method for describing the color of light generated by luminescent materials like various phosphors doped with rare earth. To obtain the CIE coordinates, the spectral distribution of the emitted light is examined, and the relative intensities of different light wavelengths are measured.

### 3.6. (CIE) Coordinates Y_2_SiO_5_: Eu^3+^

**Figure 8:**
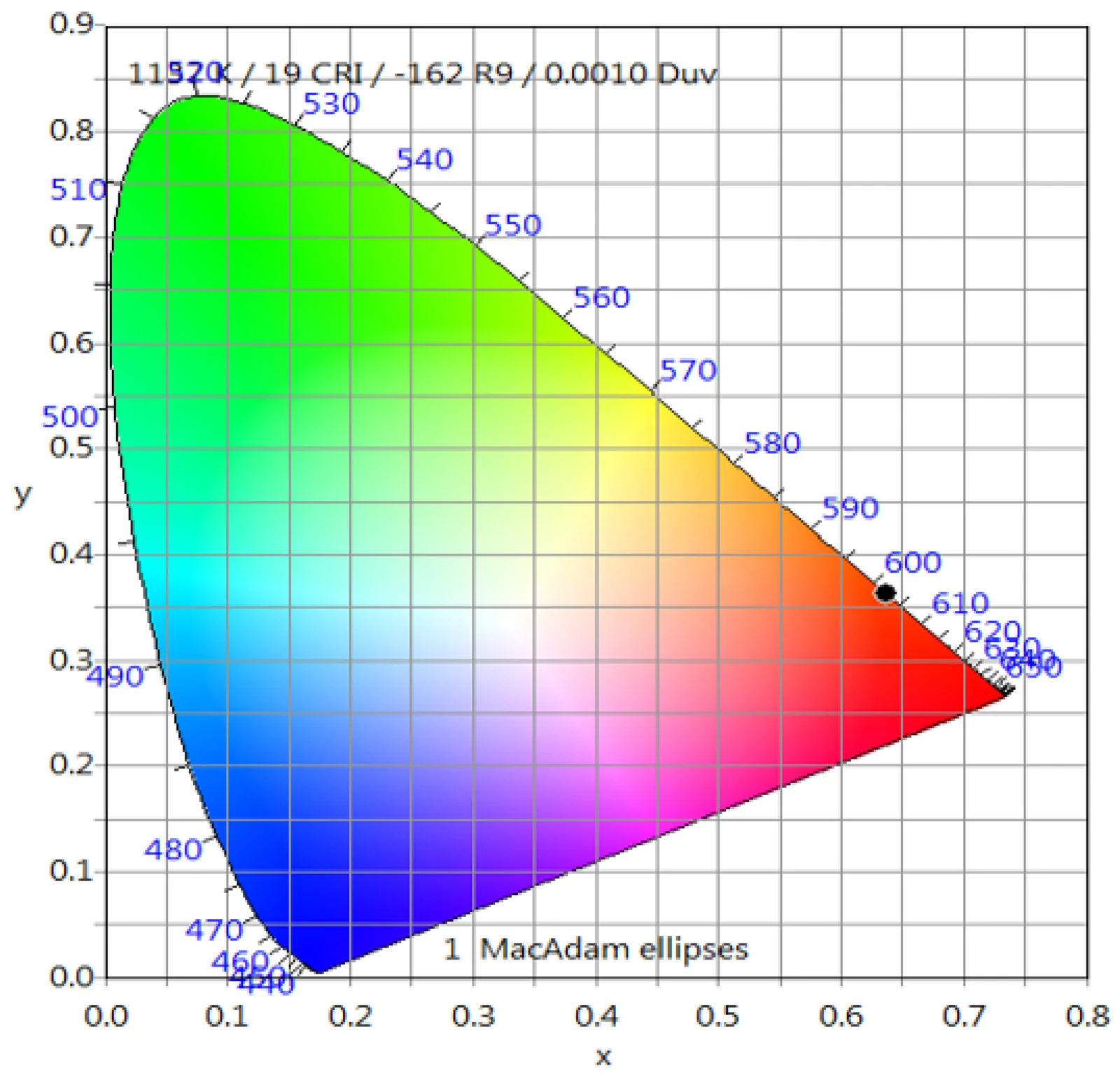
CIE coordinate for Y_2_SiO_5_: Eu^3+^ phosphor (x = 0.6363, y = 0.3633).

Y_2_SiO_5_ phosphors that have been doped with Eu^3+^ often display near-red area luminescence with CIE coordinates (Figure 8). The exact CIE coordinates could vary depending on the specific makeup and production conditions of the phosphor. The color coordinates correspond to the sample with a europium concentration of 2.0 mol%, as this concentration exhibits the highest intensity in the photoluminescence spectra.

### 3.7. Thermoluminescence Study of Y_2_SiO_5_: Eu^3+^

**Figure 9:**
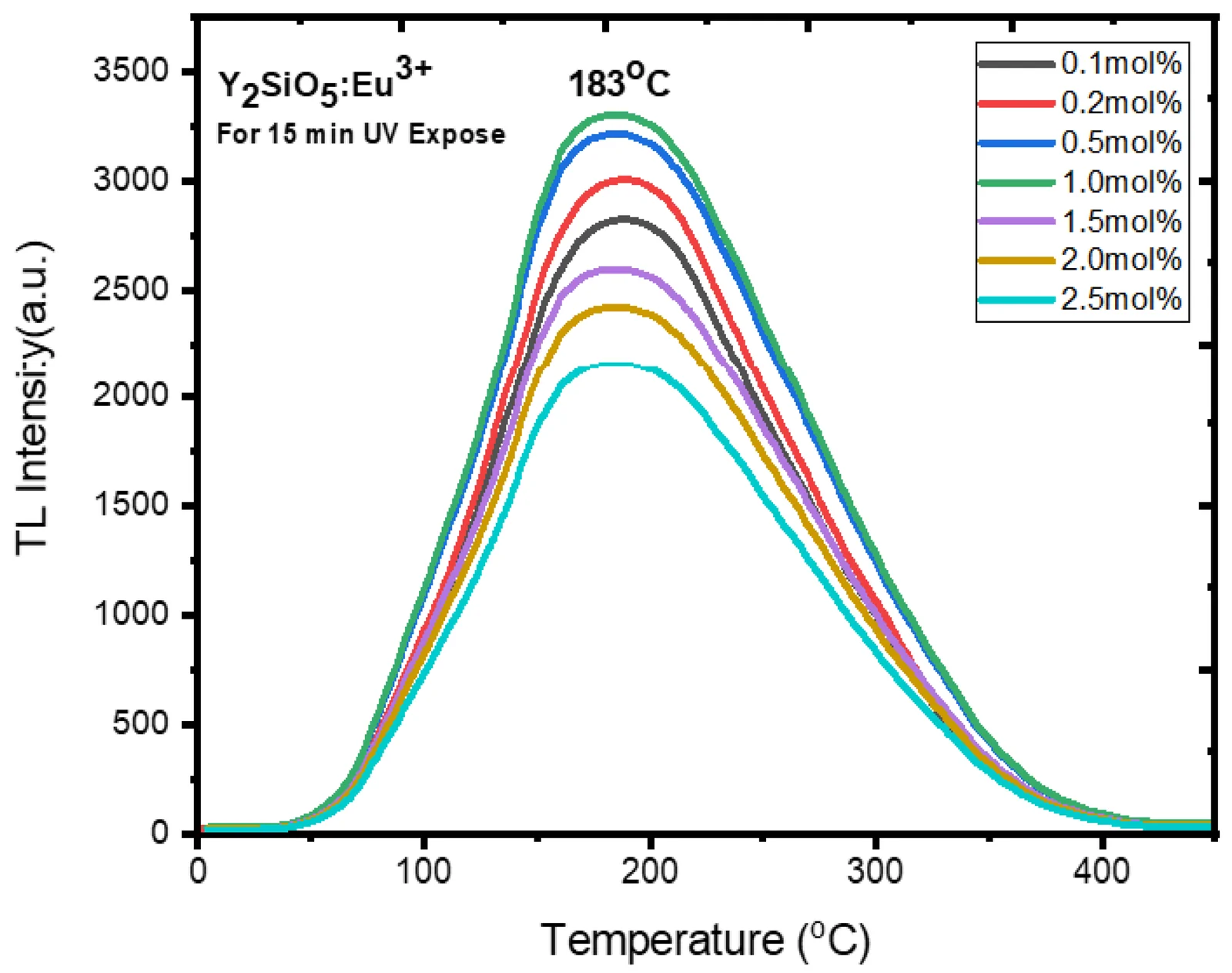
TL glow curve analysis of Y_2_SiO_5_: Eu^3+^ for all concentrations.

**Figure 10:**
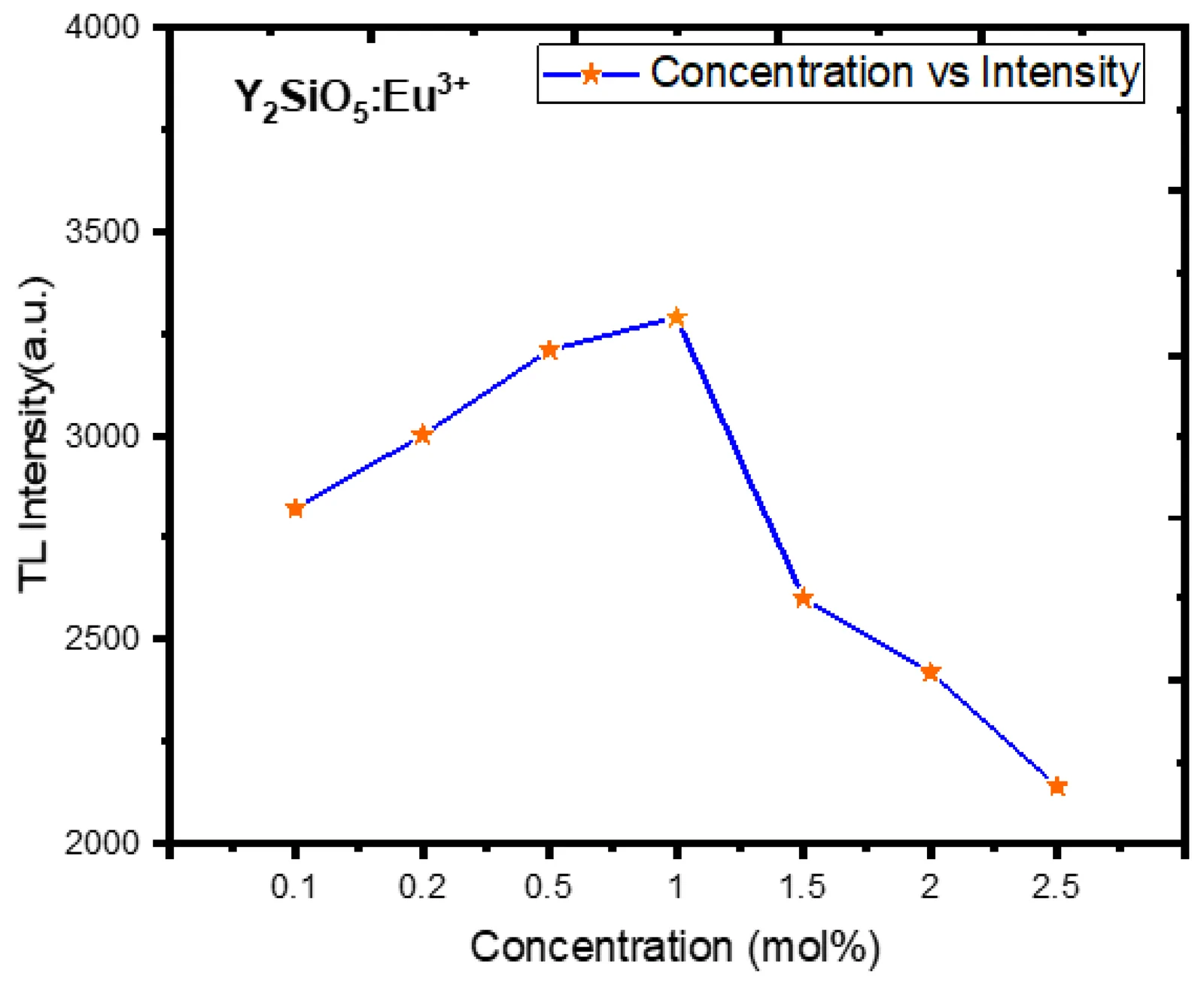
Y_2_SiO_5_: Eu^3+^ concentration vs. intensity for all concentrations.

**Figure 11:**
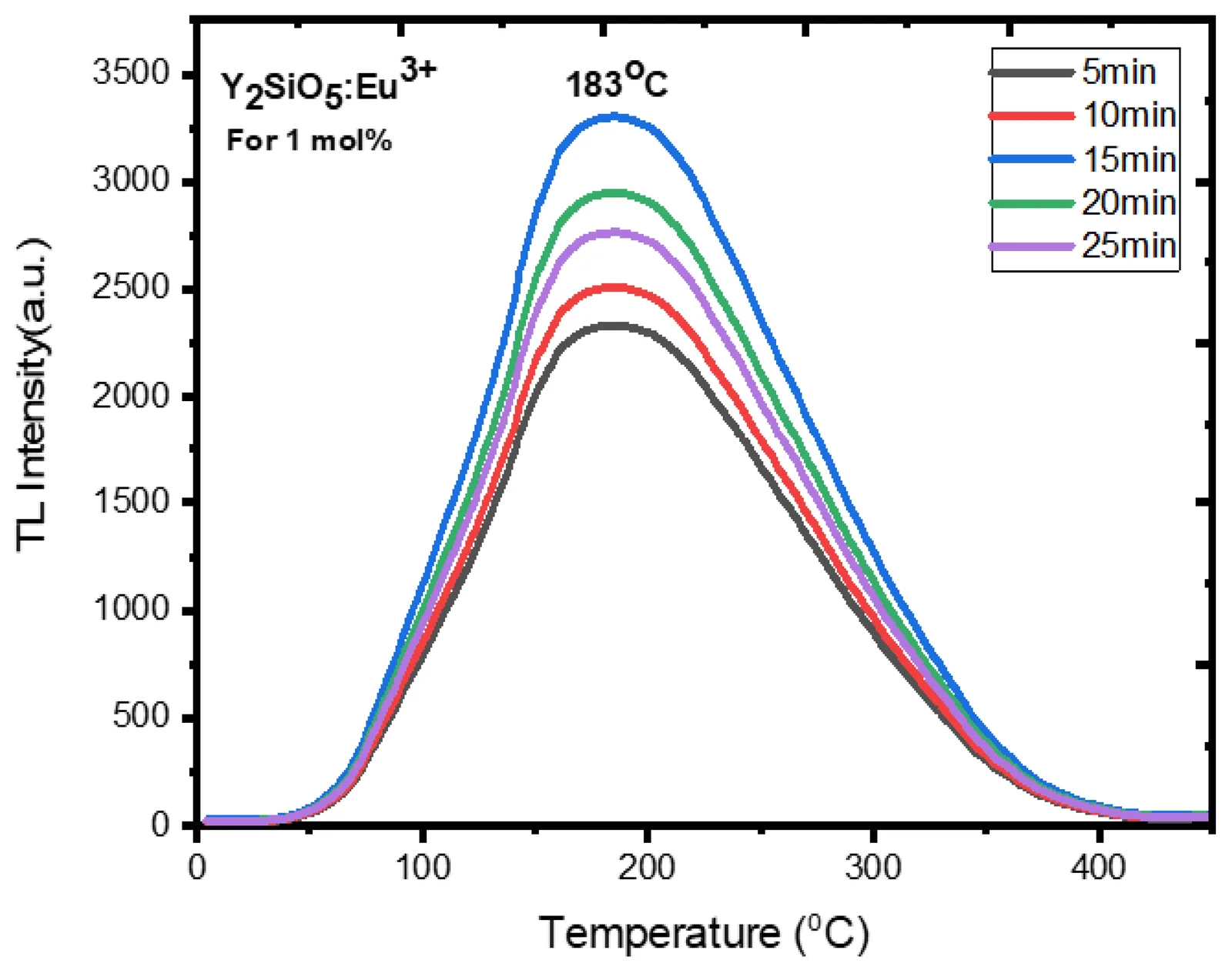
TL glow curve analysis of Y_2_SiO_5_: Eu^3+^ for 1.0 mol%.

**Figure 12:**
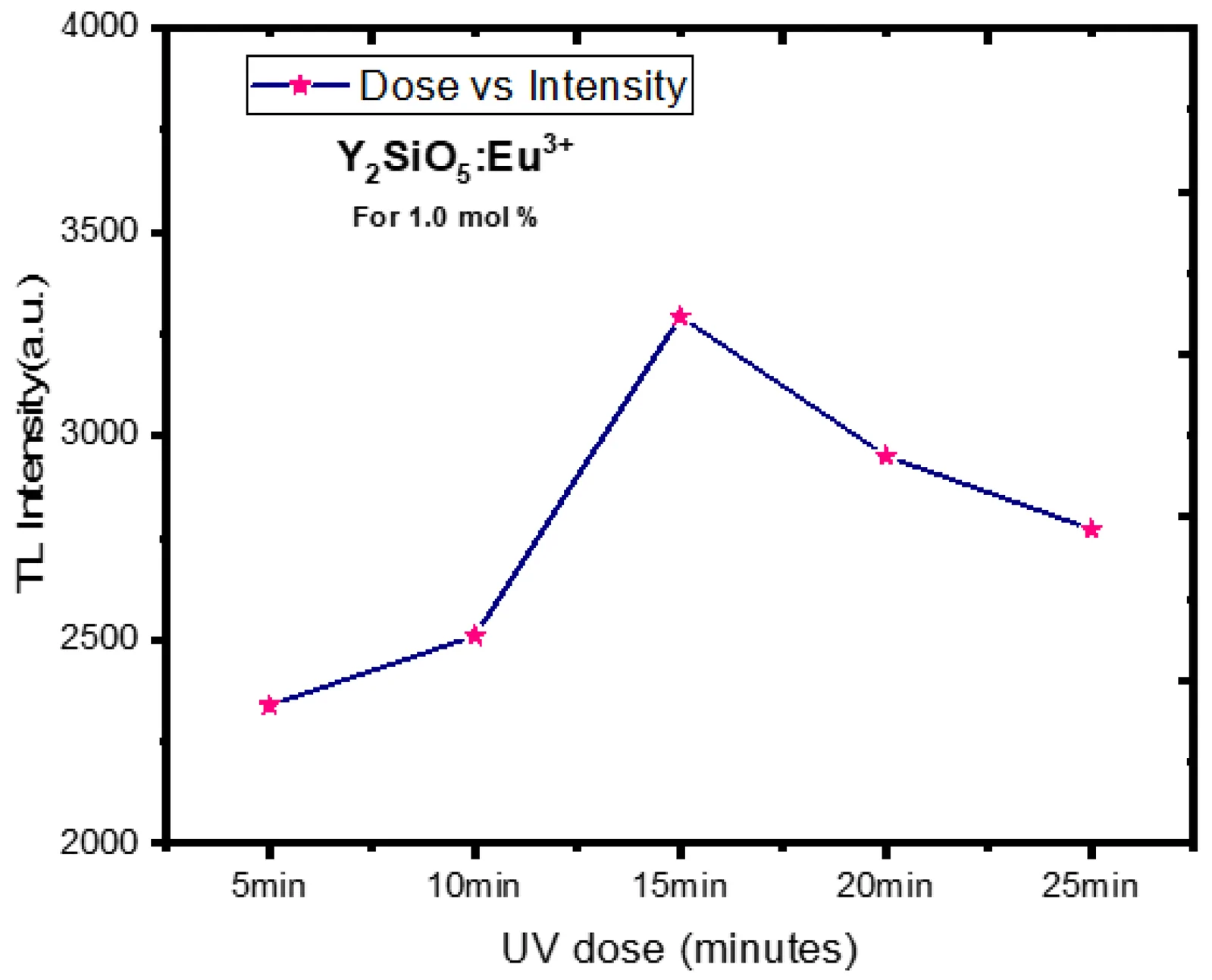
Y_2_SiO_5_: Eu^3+^ dose vs. intensity for 1.0 mol%.

The thermoluminescence (TL) study of Y_2_SiO_5_:Eu^3+^ phosphor, as shown in Figure 9, reveals a linear TL glow curve after 15 min of UV exposure. The intensity of TL increases as the concentration of europium goes up, showing a wide peak at approximately 183 °C, at which point electrons trapped in lattice defects are released. This peak is ideal for thermoluminescence dosimetry applications. The TL intensity increases up to 1.0 mol% of Eu^3+^; however, beyond this concentration, it decreases due to concentration quenching, as illustrated in Figure 10. Figure 11 exhibits the TL glow curve for the most effective 1.0 mol% concentration. It illustrates a wide peak under different UV exposure durations while maintaining a constant heating rate of 2.5 °C/s. As depicted in Figure 12, following 15 min of UV exposure at the ideal concentration, the TL intensity decreases as a result of concentration quenching.

### 3.8. Kinetic Parameter Calculation

When charge carriers (holes or electrons) are released, thermoluminescent dosimeter (TLD) phosphors typically show multiple peaks in their emission spectra. The dosimetric properties of these materials are strongly influenced by kinetic parameters, namely the activation energy (E), kinetic order (b), and frequency factor (s). These parameters provide significant insights into phosphor’s emission process. Understanding these kinetic parameters is crucial for designing effective TLDs. Chen’s empirical equations [26,27,28,29,30,31,32,33,34,35,36,37] can be used to estimate these parameters. According to Chen, the symmetry factor is

μg=δω=T2−TmT2−T1,

where T_m_ is the temperature corresponding to the maximum intensity (I_m_) of the TL peak; T_1_ is the temperature at the low-temperature side of the peak, where the intensity is half of I_m_; and T_2 is_ the temperature on the high-temperature side of the peak, where the intensity is also half of I_m_.

The activation energy (E), also referred to as trap depth, indicates the energy required for trapped electrons or holes to escape from defects within the material after irradiation. This parameter is vital because it affects the retention and eventual loss of stored dosimetric data in the phosphor. The kinetic order (b) defines how free charge carriers recombine with their counterparts, determining the type of recombination process taking place. Lastly, the frequency factor (s) represents how frequently an electron collides with the potential well’s walls within the trap, influencing the likelihood of escape. Together, these trapping characteristics form the foundation for accurate thermoluminescent dosimetry [38,39,40,41,42,43,44,45].

### 3.9. Kinetic Parameter Calculation of Y_2_SiO_5_: Eu^3+^

**Figure 13:**
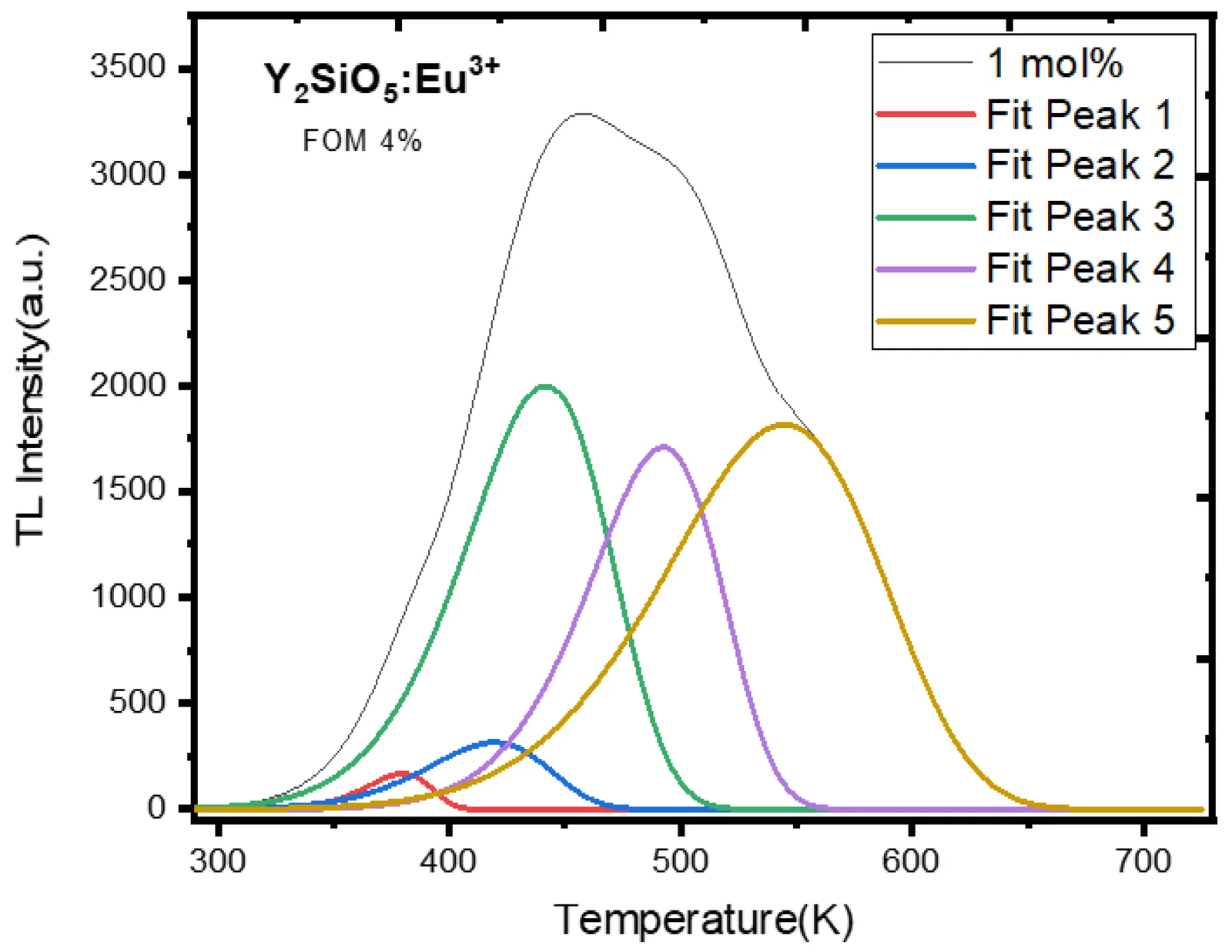
Optimum UV dosage and 1.0 mol% concentration produce CGCD pattern in UV-induced Y_2_SiO_5_: Eu^3+^-doped phosphor.

Glowfit software 3.0 was utilized for analyzing the glow curve of Y_2_SiO_5_: Eu^3+^ to apply the peak shape technique, as illustrated in Figure 12. This technique, also referred to as Chen’s peak method, aids in determining the kinetic parameters of the glow peaks for thermoluminescent materials. The CGCD pattern (Figure 13) clearly displays five broad peaks, and the corresponding kinetic parameters can be found in Table 3. The obtained glow curves of the developed phosphors exhibit a wide peak after exposure to UV irradiation for 15 min at varying concentrations of europium ions. This indicates that the phosphors are likely to be composite in composition. To determine the kinetic parameters, the CGCD method was used to deconvolute the data. Glowfit was used with the CGCD approach to deconvolute the glow curves of the highest intensity peak, which was achieved by exposing 1.0 mol% europium ions to UV light for 15 min. The flow of charge carriers between energy levels during trap emptying induced by thermal heating is described by the Halperin and Barner formulae in this program. The kinetic parameters at the trap level, such as trap depth (E) and frequency factor (s), were established for every separated peak in the thermoluminescence (TL) glow curve. The observed glow curves were compared to the glow curves generated theoretically, and the accuracy of the fit was evaluated using the figure of merit (FOM). A good fit is indicated when the FOM is less than 5%. Recent studies have shown an FOM of 4.00%, demonstrating a strong match between the theoretical predictions and the observed glow curves [44,45]. Figure 13 shows the TL glow curves for the fitted TL, while Table 3 summarizes the estimated trap depths (E) and frequency factors (s) for the trapped charges.

The value of the shape factor between 0.41 and 0.45 suggests general-order kinetics (b), while the substantial activation energy (s) of 0.50–0.86 eV needed to release a single electron from the trap level indicates that trap formation is quite sturdy. These results are summarized in Table 3. The number of electrons that escape each second (s), which ranges from 2.76 × 10^9^ to 6.21 × 10^9^ s^−1^, is also very large.

## 4. Conclusions

It is concluded that the synthesized phosphor YSO: Eu^3+^ shows a monoclinic structure and that the surface morphology was agglomerated, as confirmed by the SEM images. FTIR shows the formation of Y_2_SiO_5_ phosphor doped with Eu^3+^, and stretching and bending of Y-O and Si-O are verified. The emission spectra for photoluminescence were recorded at 582 nm and 589 nm, which correspond to the ^5^D_0_→^7^F_1_ (magnetic dipole) transition, and at 601 nm, 613 nm, and 632 nm, which correspond to the ^5^D_0_→^7^F_2_ (electric dipole) transition. These measurements were taken under 263 nm excitation. It is important to note that the electric dipole transition is more dominant than the magnetic dipole transition. Because Eu^3+^ is naturally bright, these emission peaks show how ions change from one excited state to another. This makes them useful for making phosphors that emit red light for use in optoelectronics and flexible displays. Based on the computed (1931 CIE) chromaticity coordinates for the photoluminescence emission spectra, it was determined that the produced phosphor may be used in light-emitting diodes as a red component. The TL glow curve was examined for various doping ion concentrations and durations of UV exposure, revealing a singular wide peak at 183 °C. Through the use of computerized glow curve deconvolution (CGCD), we derived the kinetic parameters. The kinetic parameters obtained suggest the formation of less deep traps upon exposure of the phosphor to UV light, with predominantly first-order kinetics observed across the glow curve.

## Figures and Tables

**Table 1:** Crystallite size determined by Scherrer’s formula.

Peak Position (2 theta)	FWHM	Crystallite Size D (nm)	Average Size D (nm)
23.33	0.202	40.201	30.824
29.667	0.163	50.388	
31.125	6.108	1.350	
41.661	0.512	16.606	
49.021	0.152	57.279	
53.018	1.360	6.25	
58.088	0.209	43.423	

**Table 2:** Powder mapping EDX spectra of Y_2_SiO_5_: Eu^3+^ phosphor (2.0 mol%).

Element	Line Type	wt%	Atomic%
O	K series	30.18	65.7
Si	K series	8.55	10.6
Y	L series	59.47	23.3
Eu	L series	1.81	0.4
Total		100	100

**Table 3:** Kinetic parameter calculation for UV-induced Y_2_SiO_5_: Eu^3+^-doped phosphor using the CGCD program.

T_1_ (K)	T_m_ (K)	T_2_ (K)	t	d	w	m = d/w	Activation Energy E (eV)	Frequency Factor s (s^−1^)
360	380	394	20	14	34	0.41	0.86	2.76 × 10^9^
283	419	447	36	28	64	0.43	0.54	2.22 × 10^9^
399	441	475	42	34	76	0.44	0.50	3.39 × 10^9^
452	492	523	40	31	71	0.43	0.68	4.21 × 10^9^
482	544	595	62	51	113	0.45	0.5	6.21 × 10^9^

## Data Availability

Data are contained within the article.
