# Correction: Verma et al. Investigation of Spectroscopic Parameters and Trap Parameters of Eu^3+^-Activated Y_2_SiO_5_ Phosphors for Display and Dosimetry Applications. *Molecules* 2025, *30*, 108

**DOI:** 10.3390/molecules31213852

**Published:** 2026-10-26

**Authors:** Neeraj Verma, Marta Michalska-Domańska, Vikas Dubey, Tirath Ram, Jagjeet Kaur, Neha Dubey, Shireen Aman, Ovica Manners, Janita Saji

**Affiliations:** 1 Department of Physics, Government G.S.G.PG. College, Balod 491226, Chhattisgarh, India; neerajphys14@gmail.com; 2 Department of Physics, Government Vishwanath Yadav Tamaskar Post Graduate Autonomous College, Durg 491001, Chhattisgarh, India; tirathsinha@gmail.com (T.R.); jagjeet_62@yahoo.co.in (J.K.); tiwarineha1441@gmail.com (N.D.); 3 Institute of Optoelectronics, Military University of Technology, 00-908 Warsaw, Poland; 4 Department of Physics, North-Eastern Hill University (NEHU), Shillong 793022, Meghalaya, India; amanshireen123@gmail.com (S.A.); ovicamanners@gmail.com (O.M.); 5 Department of Science and Humanities, School of Engineering and Technology, CHRIST University, Bangalore 560029, Karnataka, India; janita.saji@christuniversity.in

## Error in Figure

In the original publication [1], the SEM images presented in Figure 3 for the Y_2_SiO_5_:Eu^3+^ (2.0 mol%) phosphor were incorrect. The corrected Figure 3 appears below. The corrected Figure 3 contains SEM micrographs recorded at 1500×, 3500×, 7000× and 10,000× magnifications. The authors state that the scientific conclusions are unaffected. This correction was approved by the Academic Editor. The original publication has also been updated.

## Figures and Tables

**Figure 3:**
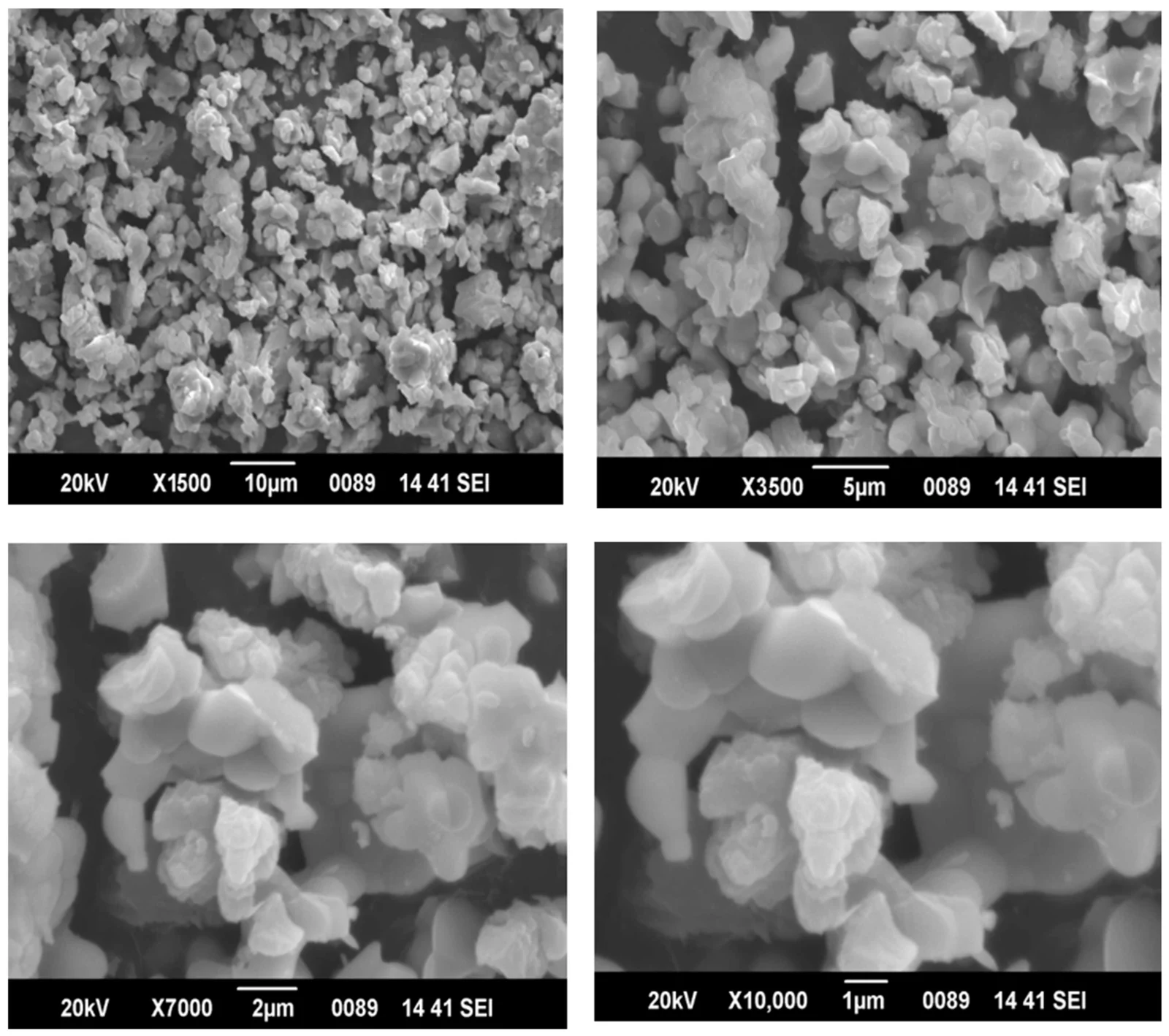
SEM images of Y_2_SiO_5_: Eu^3+^ (2.0 mol%).
